# I sync, therefore I am: brain–body synchrony in typical and disordered consciousness

**DOI:** 10.1093/nc/niag028

**Published:** 2026-06-27

**Authors:** Asa Young, Marissa Ericson, Jonathan W Schooler

**Affiliations:** Department of Psychological and Brain Sciences, University of California Santa Barbara, 551 UCEN Road, Santa Barbara, CA 93106, United States; Department of Psychology, University of Southern California, Seeley G. Mudd Building, 3620 McClintock Ave, Los Angeles, CA 90089, United States; Department of Psychological and Brain Sciences, University of California Santa Barbara, 551 UCEN Road, Santa Barbara, CA 93106, United States

**Keywords:** consciousness, embodiment, resonance, interoception, cross-frequency coupling, binding and multisensory integration

## Abstract

Consciousness is often treated as a property of the brain alone, yet accumulating evidence suggests that it is shaped by a recurrent dialogue between the brain and body. In this review, we discuss how cognition associated with consciousness—processes that define the observer, their observations, and arising qualia—depend on structured rhythmic correspondences between bodily rhythms and brain signals. We first review how cardiac, respiratory, and gastric rhythms modulate neuroelectric and neurovascular fluctuations in support of interoception—perception of the bodily state—and functionally related faculties such as emotion and selfhood, which draw upon interoceptive representations. We then discuss how clinical and subclinical conditions are associated with dysrhythmia in underlying bodily rhythms and atypical increases or decreases in a bodily rhythm’s modulation of brain processes. Across bodily systems, an emergent pattern suggests both an optimal window of brain–body synchrony, beyond which adverse effects are likely to occur, and that non-clinical, stress-related increases in brain–body synchrony may promote the utility of bodily rhythms for coordinating disparate entrained cognitive systems to facilitate functions such as multisensory integration. We conclude that these converging lines of research support a categorical difference between an embodied agent and the proverbial brain-in-a-vat because disturbing bodily rhythms or their coupling to the brain alters phenomenological experience—at times to such a degree as to warrant labeling the resulting states as meeting clinical criteria.

‘I *know*,’ this patient says, ‘that I have a heart but I do not feel it beat, except sometimes very faintly.’ When an event happens which ought to affect it, he fails equally to feel it. He does not feel himself breath, or know whether he makes a strong or weak inspiration. ‘I do not feel myself alive,’ he says.

– P. Sollier quoted by William James, *The Physical Basis of Emotion*

The proverbial brain-in-a-vat could very well be conscious (Trujillo et al. 2019; Lavazza 2021; Sharf et al. 2022). It is conventional to situate the seat of subjective experience within the physiology of the brain and often in specific regions rather than its totality^1^ (Northoff and Lamme 2020; Seth and Bayne 2022; Cogitate Consortium et al. 2025). To challenge the historical isolation of the observer to the confines of the skull, embodied and enactive neuroscience asserts a firm and irreducible embedding of cognitive processes in the somatic environment that sustains and stimulates the nervous system (Thompson and Varela 2001; Fuchs 2009; Foglia and Wilson 2013). Indeed, this view has led some to argue that a vatted brain, severed from the body’s reentrant loops, would lack the necessary biological underpinnings to experience any consciousness altogether (Thompson and Cosmelli 2011).

The contention that consciousness could reside within the proverbial brain-in-a-vat motivates an examination of our brain’s relationship with its body. Consciousness may be understood as a constellation concept: not a single process, but a family of overlapping phenomena including, among other faculties, selfhood, perception, and emotion. We therefore approach consciousness by way of these constituent processes, which define the observer, the contents of their observation, and the subjective feelings born of these contents. We argue that bodily states are a necessary component of conscious states, by discussing, first, the role of the body in constraining cognition commonly associated with consciousness and, second, how disruptions to brain–body integration precipitate disruptions to conscious states—to such a degree in some cases as to warrant labeling these states as meeting clinical criteria.

Kluger et al. (2024) frame the perspective that cognitive neuroscientific research can be simplified from a near-infinite information space to a set of constrained trajectories that are determined by the inner and outer environment. Contemporary views of functionally relevant neural organization describe a hierarchical arrangement of high-frequency, cortically generated rhythms nested within comparatively lower-frequency, network-level rhythms (Lakatos et al. 2005). Embodied accounts extend this nesting thesis to include the lower timescales of organismal processes as functionally relevant scaffolds for merging neural networks responsible for various cognitive processes with bodily activity responsible for maintaining homeostasis (Klimesch 2018; Azzalini et al. 2019; Young et al., 2022a; Criscuolo et al. 2022). That is, our body—the visceral and somatic environment—wields a considerable influence over our cognition and perception through a recurrent modulation of brain processes.

There are several extant perspectives that describe the body’s contributions to conscious states and, further, to specific aspects of consciousness and cognition. The neural subjective frame is a model that proposes that afferent signals are collated into a low-level representation of the physical self from which the first-person perspective is born (Park and Tallon-Baudry 2014; Babo-Rebelo and Tallon-Baudry 2018). In this model, bodily information is a necessary building block of the “I” in the statement “I hear/see/smell/feel/taste something.” Predictive coding frameworks propose a bidirectional exchange of bottom-up afferent signals and top-down predictions whereby the brain is supplied with updates to internal models and models are acted upon to align outcomes with predictions (Friston 2010; Seth 2013)—a dynamic theorized to emerge as early as gestation (Corcoran et al., 2025a, 2025b). The perspective reframes the James–Lange theory of emotion (James 1894) to emphasize “inference” over “appraisal,” suggesting instead that emotional states result from the active inference of internal and external causes of physiological change (Seth 2013). Dynamical systems frameworks, such as the neurovisceral integration model (Thayer and Lane 2000; Smith et al. 2017), propose that organisms constitute a collection of loosely coupled oscillators whose activity and variation are regulated in feedback loops that guide emotional and homeostatic regulation.

The varying perspectives, only a few of which were highlighted here, differ in some aspects of their models, but together map the theoretical landscape regarding the functional role of bodily states in conscious states. The functional role is, first, that of interoception—the ability to monitor, perceive, and interpret the internal state of the body (Craig 2003; Chen et al. 2021). This functional role extends to viscerally grounded cognition, such as emotion and selfhood, which implicitly and explicitly involves interoceptive representations (Azzalini et al. 2019). Some frameworks extend the functional role to decision-making under uncertainty, such as the somatic marker hypothesis (Bechara et al., 1994), in which visceral signals bias decision-making often beyond awareness. Others extend it to empathy, suggesting that the mental representations we generate of others are extrapolated from the interoceptive representation of the self (Ondobaka et al. 2017; Palmer and Tsakiris 2018), and more recently to time perception, in which bodily rhythms distributed across slower spatiotemporal scales provide mechanisms for temporally coordinating neural events to experiential moments (Buzsáki 2025).

Interoception, exteroception, emotion, and selfhood are products, in part, of the binding of brain and bodily systems that generate a biologically implemented point of view from which we are conscious and populate this point of view with qualia that contain a rich visceral dimension (Park and Tallon-Baudry 2014). To unbind these systems, wholly or partially, would in theory dissolve the cognitive faculties that are products of their union proportional to the disturbance. Developmental, neurodegenerative, and psychogenic disorders are often comorbid with bodily dysfunction (Sartorious 2013). Hyperventilation can spur panic (Sikter et al. 2007); anxiety disorders are associated with decreased heart rate variability (HRV) and increased risk of cardiovascular disease (Chalmers et al. 2014); traumatic stress is commonly concomitant with functional gastrointestinal disorders (Kolacz et al. 2019). The pattern of brain-to-body and body-to-brain dysfunction gives rise to the popular sentiment that mental illness is not solely mental nor is bodily disorder relegated to the viscera.

Our discussion will be that of rhythms—of how bodily cycles such as the heartbeat, breath, and gastric basal rhythm modulate brain activity *via* structured rhythmic correspondences of temporally aligned frequency, phase, and amplitude. Among the measurable correlates of consciousness (Crick and Koch 1990), we will evaluate embodied cognition through the lens of the oscillatory correlates of consciousness, wherein the dynamics of the nervous system’s rhythmic architecture are unified with the dynamics of phenomenal consciousness (Gallotto et al. 2017; Hunt et al. 2022), particularly when those rhythmic dynamics go awry and phenomenal consciousness is altered. By coordinating rhythmic activity, the information content and complexity of distributed systems, such as the brain and body, may be effectively functionally unified in time despite their separation in space (Young et al., 2022a). Synchrony, as a means to overcome the binding problem, places a premium on the phenomenon in neuroscientific research (Buzsáki 2006; Canolty and Knight 2010; Fries 2015) and makes it a cornerstone of some theories of consciousness (Grossberg 2017; Hunt and Schooler 2019; Riddle and Schooler 2024).

Our discussion will involve three major points. First, interoception and functionally related cognition are underpinned by structured rhythmic correspondences between brain and body systems. Second, these rhythmic correspondences and the cognitions they support are vulnerable to dysfunction in the underlying bodily rhythm or the magnitude of its modulation over brain processes, contributing to the development and aggravation of neuropsychiatric disorder. Third, the degree to which disruption to bodily processes or the brain’s communication with bodily processes may alter phenomenology emphasizes the categorical difference between the embodied and vatted brains, emphatically so because to disturb embodiment is to disturb the visceral dimension implicit to conscious states.

## 1. The brain–body oscillatory hierarchy

We begin by articulating the first major point—that interoception and functionally related cognition are underpinned by structured rhythmic correspondences between brain and body systems. The heartbeat, breath, and gastric basal rhythm are organismal processes whose rhythms characterize cardiac, respiratory, and gastrointestinal systems. Recent work has demonstrated these rhythms modulate cognition by entraining underlying brain processes, functionally merging brain activity with bodily activity. The cortical and subcortical sites of rhythmic correspondence with bodily rhythms encompass nodes of many canonical resting-state networks including the default mode, saliency, and dorsal attention networks (Kluger and Gross 2021; Rebollo and Tallon-Baudry 2022). Embodied cognition in neuroscientific terms is thus two-fold in function (Tallon-Baudry et al. 2026). First, bodily rhythms convey information about the bodily state, and their phase may modulate neural excitability in ways that contribute to interoception, affective inference, and homeostatic regulation. Second, the phasic nature of bodily rhythms may impose rhythmic constraints on spontaneous and functionally relevant neural activity, thereby facilitating the coordination of distinct cognitive systems by virtue of their shared entrainment (Azzalini et al. 2019; Engelen et al. 2023). A proposed consequence of this latter function is that these shared temporal constraints may contribute to the unity of perceptual experience by aligning distributed sensory and cognitive processes according to a common egocentric coordinate system (Engelen et al. 2023; Tallon-Baudry et al. 2026). The following will provide an overview of the putative rhythmic correspondences between the heart, breath, gut, and brain as well as frameworks that bridge rhythmic correspondences between systems.

### 1.1 Heart–brain rhythmic correspondence

Beating 100 000 times a day and 2.5 billion times in a lifetime (Shaffer et al. 2014), the heart was once considered by ancient scholars to be the seat of consciousness (Jaynes 1976; Cobb 2020). It is the most powerful generator of rhythmic information within the body (McCraty et al. 2009), an intrinsic rhythm that is modulated top-down by the central autonomic nervous system but would continue to beat despite severance from the brain (Thayer et al. 2012).

The heartbeat is characterized by two phases: systole, the muscle contraction phase; and diastole, the relaxation phase (Candia-Rivera et al. 2026). The phasic nature of the heartbeat constrains interoceptive and exteroceptive cognition as described in such accounts as the baroreceptor hypothesis^2^ (Lacey and Lacey 1970), which suggests that maximal baroreceptor activation at systole recurrently inhibits cortical excitability, with implications for perceptual processes. Processing of nociceptive (Edwards et al. 2001), auditory (Tanaka et al. 2023), visual (Salomon et al. 2016), and tactile stimuli (Al et al. 2020; Grund et al. 2022) is attenuated when presented at systole relative to diastole. Similarly, items encoded at systole are recalled less accurately than items encoded at diastole (Garfinkel et al. 2013). Cardiac cycle effects, though, can also be facilitative: distractors presented at systole are more effectively suppressed (Pramme et al. 2014; Pramme et al. 2016) and affective stimuli are rated as more intense, capture attention more strongly, and gain faster access to awareness when presented during systole (Gray et al. 2012; Garfinkel et al. 2014; Leganes-Fonteneau et al. 2021; Ozturk et al. 2025).

Skora et al. (2022) proposed that the aforementioned cardiac cycle effects and the reflexive cardiac deceleration to anticipated stimuli reflect a gain modulation of interoceptive and exteroceptive attention. The recurrent interoceptive signal at systole can interfere with concurrently presented exteroceptive stimuli; anticipatory or error-related cardiac deceleration reduces the frequency of baroreceptor signals and increases the relative precision afforded to external sensory evidence. Facilitative effects, notably enhanced processing of emotional facial expressions, suggest that cardiac phase may interact differently with exteroceptive stimuli that draw on interoceptive representations. One provisional interpretation is that maximal baroreceptor signaling at systole suppresses exteroceptive inputs that compete with cardiac afference, while increasing the relative weight of exteroceptive cues that can be integrated with concurrent interoceptive representations. This integrative account may help explain why nociceptive and neutral sensory stimuli are attenuated during systole, whereas affective stimuli may be facilitated. Consistent with this interpretation, exteroceptive–interoceptive multisensory integration appears to be enhanced at systole, as evidenced by the facilitation of the rubber hand (Suzuki et al. 2013), full-body (Aspell et al. 2013), and enfacement illusions (Sel et al. 2017) when incorporating systolic-locked cardio-visual feedback. In contrast, exteroceptive–exteroceptive multisensory integration (i.e. tactile-visual and tactile-auditory) is enhanced at diastole relative to systole (Saltafossi et al. 2023).

In short, cognition is modulated by the phasic nature of bodily rhythms such as the heartbeat, and visceral information is continuously integrated into perception and cognition—two motifs that recur throughout this article. The following will review research regarding the modulation of neural processes by the heartbeat, as well as by the rate and variability of its occurrence.

#### 1.1.1 Heartbeat-evoked potentials

The heartbeat evokes a cortical response, known as the heartbeat-evoked potential (HEP), a scalp-recorded event-related potential time-locked to the R-wave of the electrocardiogram and commonly interpreted as a reflection of cortical processing of cardiac afference (Schandry et al. 1986). Although the latency and spatial distribution of the HEP vary across studies, effects are commonly observed 200–600 ms following the R-wave, with meta-analytic effects often emerging over central and fronto-central electrodes (Coll et al. 2021). A two-component HEP model has been proposed in which the early component (100–250 ms) reflects interoceptive processing of the heartbeat and the late component (250–500 ms) reflects a context-dependent elaborative process (Gautier et al. 2025). The magnitude of the early-latency HEP is associated with inter-individual differences in heartbeat-counting accuracy (Pollatos and Schandry 2004) and increases with physiological arousal (Coll et al. 2021), whereas the magnitude of late-latency HEP increases with interoceptive attention (Petzschner et al. 2019; Coll et al. 2021). Moreover, the HEP is sensitive to thought content that draws upon interoceptive representations. For example, the magnitudes of early and late HEP components at central and fronto-central channels are increased by engaging in empathic tasks (Fukushima et al., 2011), while the magnitude of the late-latency HEP at posterior sites is increased during imagination regarding the self (Babo-Rebelo et al. 2019).

Intracranial evidence indicates that the HEP is best explained by a phase-resetting of spontaneous theta-band activity 100–250 ms following the R-wave, primarily within the insula and operculum, but also in the amygdala and distributed fronto-temporal regions (Park et al. 2018). In a full-body illusion paradigm, modulation of late-latency HEP within the insula was observed in patients who reported stronger self-identification with an avatar during synchronous relative to asynchronous visuo-tactile stimulation (Park et al. 2018). Together, the magnitude of the HEP appears to reflect interoceptive processing of cardiac afferents and higher-order processes which may draw upon interoceptive representations.

#### 1.1.2 Heart–alpha cross-frequency synchronization

The heart rate is an informative cue to the state of the cardiovascular system, varying with states (e.g. accelerating with arousal) and traits (e.g. lower in males). A recent theory of brain–body coupling, to be discussed in greater detail in following sections, proposed that transient alignment of frequency between neuroelectric frequency bands (i.e. EEG) and cardiorespiratory rhythms may facilitate cross-frequency phase–phase synchrony (cross-frequency synchronization; Palva and Palva 2018) within and between brain–body systems (Klimesch 2018). This form of coupling between occipital alpha frequency and heart rate is enhanced as a function of physiological arousal, effectively decoupling during sleep (Rassi et al. 2019). The sleep-related decoupling, consistent with the reduction of late-latency fronto-central HEP amplitude with increasing sleep depth (Lechinger et al. 2015), is conjectured to reflect the recession of the heart’s phasic modulation of cognitive processes and, in particular, bodily awareness during sleep states (Klimesch 2023). Coupling between occipital alpha frequency and heart rate is also enhanced as a function of cognitive demand and, further, the directionality of coupling is ascending (Soriano et al. 2024). Both empirical studies, consistent with their motivating theory (Klimesch 2018), suggest that this form of coupling between the alpha band and heart rate may index transitions from low-to-high demand states that involve fluctuations in bodily awareness.

#### 1.1.3 HRV–neuroelectric phase-amplitude coupling

Heart rate variability (HRV) is an emergent property of the heart rate, reflecting the fluctuations in the interval between consecutive heartbeats (McCraty et al. 2009; Shaffer et al. 2014). It is commonly interpreted as a biomarker reflecting autonomic regulation: parasympathetic activation inhibits the intrinsic heart rate and lengthens the interbeat interval, while sympathetic activation disinhibits the intrinsic heart rate and shortens the interbeat interval (Thayer et al. 2012). The HRV spectrum includes a low-frequency component (0.04–0.15 Hz; LF-HRV) that is generated by baroreceptor-related regulation by both branches of the autonomic nervous system and a high-frequency component (0.15–0.4 Hz; HF-HRV) that corresponds to vagally mediated parasympathetic regulation closely associated with respiratory-related heart rate fluctuations (Shaffer and Ginsberg 2017). Healthy cardiorespiratory systems are characterized by relatively high variability, namely, greater power in the HF-HRV range, allowing the organism to adaptively respond to exogenous and endogenous influences (Chalmers et al. 2014).

Robust associations between HRV and positive mental (Appelhans and Luecken 2006; Ottaviani et al. 2016) and physical outcomes (Chalmers et al. 2014) motivated the hypothesis that HRV may constitute an oscillatory signal that can enhance functional connectivity between regulatory networks by inducing synchronization in prefrontal neural oscillations (Mather and Thayer 2018). Consistent with the hypothesis, it was demonstrated that the phase of HF-HRV modulates the amplitude of all five characteristic frequency bands of fronto-central EEG, with Granger causality indicating a predominant heart-to-brain directionality (Sargent et al. 2024).

Phase-amplitude coupling is not limited to HF-HRV. Xiong et al. (2025) found that the phase of LF-HRV modulated the amplitude of low-frequency delta and high-frequency beta and gamma EEG over prefrontal and central channels, and that this modulation was enhanced by slow breathing exercises and associated with emotional control. Liao et al. (2025) reported that LF-HRV phase modulated right frontal theta- and fronto-temporal-to-central alpha-band amplitude, and source-localized LF-alpha coupling to bilateral insula and left entorhinal cortex. As with HF-HRV, Granger causality analyses indicated that LF-HRV phase-amplitude coupling was likewise characterized by heart-to-brain directionality.

### 1.2 Breath–brain rhythmic correspondence

The breath is the bodily rhythm we most commonly, consciously interface with. Human respiration, occurring at an average frequency of 0.2–0.33 Hz in healthy adults (Scott and Kaur 2020), is maintained autonomously by the pre-Bötzinger complex in the medulla, but may be exercised with volition through cortical projections that influence the respiratory network (Brændholt et al. 2023). Breath is employed, first, for blood oxygenation, but also for sensory sampling, communication, and volitional modulation of the heart during contemplative practice (Engelen et al. 2023).

Like the heart, the respiratory cycle appears to influence behavior by creating functionally relevant phases of perception and action. Respiration is commonly described in two phases: inspiration, during which air is drawn into the lungs; and expiration, during which air is expelled. Humans spontaneously align inspiration to trial onset, and when (notably, nasal) inspiration and stimulus presentation are experimentally or spontaneously aligned, performance is facilitated during memory retrieval, fear discrimination (Zelano et al. 2016), and visuospatial processing (Perl et al. 2019). Across a broad battery of tasks, Johannknecht and Kayser (2022) found that participants tended to align inspiration with stimulus presentation and expiration with response execution. Behavioral covariation with respiration was most evident for reaction times in pitch discrimination, sound detection, and emotion discrimination tasks.

While inspiration therefore appears preferentially aligned with sensory acquisition, expiration appears preferentially aligned with action and response execution. Consistent with this interpretation, Park et al. (2020) found that self-initiated voluntary actions occurred more frequently during expiration in voluntary-action tasks. This respiration–action coupling was absent for externally triggered actions and was accompanied by respiratory-phase modulation of the readiness potential. However, recent work suggests that respiratory phase alters decision-making rather than merely shifting overt response timing. In a visual motion and affective face discrimination task, Brændholt et al. (2025) found that responses made during inspiration were faster than responses made during expiration in both tasks. Response acceleration, though, reflected distinct mechanisms: during visual motion discrimination, inspiration reduced evidential decision boundaries, biasing participants toward speed over accuracy, whereas during affective face discrimination, inspiration shifted the starting point of evidence accumulation toward “happy” responses. Thus, respiratory phase appears to modulate not only when perception and action occur, but how sensory evidence is translated into decisions.

Also like the heart, respiration may contribute to bodily self-consciousness—the perceptual self which occupies a body in space (Blanke et al. 2015). The phase of respiration can modulate self–other discrimination: humans are better able to recognize their own voice from the voice of another during periods of inspiration (Orepic et al., 2022). Respiratory signals can also blur self–other boundaries through interoceptive–exteroceptive multisensory integration: synchronous respiratory–visual feedback can induce or enhance full-body illusions, altering self-location, breathing agency, and body ownership (Adler et al. 2014; Monti et al. 2020).

As described, respiration can modulate the precision-weighting of interoceptive, somatosensory, and exteroceptive signals, such that different phases of the breath alter which forms of evidence are afforded greater influence over perception, action, and self-related processing (Allen et al. 2023). In other mammals, respiration is well established as an organizer of neuroelectric activity, entraining rhythms in olfactory and non-olfactory regions, including the hippocampus, with functional consequences for sensory sampling behavior (Tort et al. 2018). Building on this animal literature, Heck et al. (2017) proposed that respiration contributes a continuous rhythmic component to ongoing cortical activity and may modulate the temporal organization of sensory, motor, emotional, and cognitive processes. The following will review human breath–brain coupling in its various forms.

#### 1.2.1 Breath***–***brain neuroelectric coupling

Zelano et al. (2016) found that spontaneous nasal respiration modulates intracranial oscillatory activity in olfactory and limbic regions. In piriform cortex, nasal respiration entrained slow local field potential fluctuations at the respiratory frequency and produced inspiration-locked increases in delta and theta power. Inspiration-locked modulation was also observed in amygdala, most consistently in delta power, and in hippocampus, more variably across delta, theta, and beta bands. Zelano et al. (2016) further reported that breath–brain coupling depended on nasal airflow: shifting to oral breathing reduced respiratory-locked oscillatory power in piriform cortex, amygdala, and hippocampus and abolished slow respiratory entrainment in piriform cortex. A complementary intracranial study indicated respiration-locked oscillations extended to the parietal, sensorimotor, premotor, prefrontal, cingulate, insular, and visual cortices (Herrero et al. 2018). Respiration-locked oscillations were sensitive to cognitive states: volitional acceleration of breathing increased coherence in frontal, insular, temporal, and amygdalar sites; interoceptive attention to the breath increased coherence in anterior cingulate, insular, premotor, and hippocampal sites.

Noninvasively, Kluger and Gross (2021) found that respiratory phase modulated oscillatory amplitude across all major frequency bands from 2 to 150 Hz. Source-localized respiration-modulated brain oscillations were distributed across cortical and subcortical regions, including anterior and posterior cingulate cortex, supplementary motor area, insula, frontal eye field, temporal and parietal cortices, orbitofrontal cortex, parahippocampal cortex, brainstem, and cerebellum. Respiration-modulated regions overlapped with nodes of the default mode, dorsal attention, and salience networks, as well as respiratory control networks. Converging evidence suggests that respiration phase modulates cortical excitability: parieto-occipital alpha power—an inverse marker of visual cortical excitability—and the posterior 1/f slope—an index of excitation–inhibition balance—are modulated by respiration phase (Kluger and Gross 2021; Kluger et al. 2023). As such, breath may be employed, spontaneously or volitionally, to coordinate sensory sampling to the excitability of internal networks (Corcoran et al. 2018; Allen et al. 2023; Brændholt et al. 2023), modifying perceptual processes by acting as a cyclic gain control (Varga and Heck 2017).

#### 1.2.2 Breath***–***alpha cross-frequency synchronization

Like heart rate, breathing frequency was found to couple with the frequency of occipital alpha (Rassi et al. 2019). Coupling between alpha and breathing frequency was enhanced as a function of physiological arousal and, like heart rate, effectively decoupled during sleep states—an effect conjectured to reflect reduced bodily awareness.

### 1.3 Stomach***–***brain rhythmic correspondence

The enteric nervous system—the control system of the gut—comprises ~500 million neurons, a neural mass comparable in size to the octopus nervous system (Young 1963), and is sometimes referred to as a second brain. The relationship between the central and enteric nervous systems is unique in the multimodal links that include neural, immune, metabolic, endocrine, and oscillatory connections forming what is referred to as the gut–brain axis (Jena et al. 2020). Rooted in vertebrate evolutionary history, this axis is implicated as a central component of state regulation, linking digestive function with socioemotional processes through interacting autonomic systems that coordinate homeostasis, threat responses, and affiliative behavior (Kolacz et al. 2019).

Interstitial cells of Cajal, gastrointestinal pacemakers within the stomach wall, generate an infra-slow 0.05-Hz rhythm known as the gastric basal rhythm, which regulates smooth muscle excitability and gastric motility (Sanders et al. 2014). Although the gastric basal rhythm paces smooth muscle contractions and increases in amplitude during digestion (Rebollo and Tallon-Baudry 2022), it is intrinsically generated (Rebollo et al. 2021): the rhythm persists in the absence of digestion and when the stomach is severed from the central nervous system. Recent work suggests that the gastric basal rhythm, like the heartbeat and breath, may impose cycle-dependent constraints on behavior. Loescher et al. (2026) found that reaction times on a speeded supra-threshold tactile detection task, a self-paced visual exploration task, and an unspeeded near-threshold visual detection task were idiosyncratically coupled to gastric phase, independent of cardiac and respiratory rhythms.

The gastric basal rhythm has been found to couple with neuroelectric and neurovascular signals in brain regions associated with homeostasis, vigilance, and interoception. The following will review research regarding stomach–brain coupling in its various forms.

#### 1.3.1 Gastric***–***neuroelectric phase***-***amplitude coupling

The first instance of oscillatory coupling between brain and stomach rhythms was found in the modulation of the largest component of waking EEG, the alpha band, by the gastric basal rhythm (Richter et al. 2017). Spontaneous fluctuations in the amplitude of the alpha band were modulated by the phase of gastric rhythms anteriorly in the right anterior insula and inferior frontal gyrus, as well as posteriorly in the parieto-occipital sulcus and calcarine fissure. Peaks in the amplitude of alpha, an inhibitory oscillation (Klimesch et al. 2007), coincided with troughs in gastric phase indicating gastric rhythms coordinated excitability in regions responsible for interoception and exteroception. Balasubramani et al. (2022) found that gastric–alpha phase-amplitude coupling was stronger during rest than during working memory in bilateral fronto-central clusters and a left parieto-occipital cluster. Although coupling generally decreased during working memory relative to rest, stronger left parieto-occipital gastric–alpha coupling during working memory was associated with faster task performance. The latter study, consistent with the hypothesis that stomach–brain interaction concerns feeding (Rebollo et al. 2021), conjectured that the relationship between alpha modulation and working memory performance reflected a gastric contribution to vigilance—for feeding opportunities or threats.

More recent work suggests that gastric–brain phase-amplitude coupling is not limited to the alpha band. Berther et al. (2026) reported widespread gastric modulation of spontaneous cortical oscillations across delta, theta, alpha, and beta bands at rest. Although the alpha band showed the strongest and most spatially extensive effects, gastric phase-amplitude coupling was also observed in delta, theta, and beta bands across unimodal and transmodal cortical regions, including insular, somatosensory, cingulate, prefrontal, and occipital cortices. Berther et al. (2026) further found that gastric–neuroelectric phase-amplitude coupling overlapped with the fMRI-derived gastric resting-state network that will be discussed in the following section.

#### 1.3.2 Gastric***-***BOLD phase-locking

Rebollo et al. (2018) identified a novel delayed-connectivity resting-state network whose blood-oxygen-level-dependent (BOLD) fluctuations were phase-synchronized with the gastric basal rhythm at each participant’s normogastric frequency. The gastric network encompassed regions typically considered functionally distinct, including regions associated with touch (primary and secondary somatosensory cortices), action (medial wall motor regions), and vision (the extrastriate body area), together suggesting a multimodal mapping of bodily space. A subsequent anatomical characterization showed that most gastric-locked cortex lies within unimodal systems—somatomotor, auditory, and visual cortices—with minor overlap in the default mode and saliency networks (Rebollo and Tallon-Baudry 2022). Using a complementary network-level approach, Choe et al. (2021) applied spatial independent components analysis to highly sampled resting-state fMRI data from a single participant and found that 3 of 18 ICA-derived networks—cerebellar, dorsal somatosensory-motor, and default mode networks—were significantly phase-locked to the gastric rhythm. The overlap of gastric phase-locked regions with unimodal and transmodal systems has been conjectured to support coordination among systems involved in exteroception and interoception, potentially facilitating multisensory integration by providing a shared temporal frame (Azzalini et al. 2019; Rebollo et al. 2021).

### 1.4 Inter-system frameworks

Our review of brain–body rhythmic correspondences has focused separately on the heartbeat, breath, and gastric cycles and their modulation of brain and cognitive processes. These systems, though, are not independent of one another. To name some examples, HRV is directly coupled to respiration through the respiratory sinus arrhythmia: heart rate accelerates at inspiration and decelerates at expiration (Yasuma and Hayano 2004). Saccades, which themselves entrain neural processes in visual cortex, medial temporal lobe, and thalamus (Leszczynski et al. 2021), are subject to cardiac cycle effects (Galvez-Pol et al. 2020). The same neural process (e.g. corticospinal motor excitability) can be subject to non-redundant modulation by cardiac, respiratory, and gastric rhythms simultaneously (Engelen et al. 2025). To move beyond single-system accounts, the following section discusses frameworks that view brain–body interaction through the lens of nested, coupled rhythms whose time-varying coordination within and between bodily and neural systems reflects and supports cognitive states.

#### 1.4.1 Network physiology

In the emerging field of network physiology, the human body is modeled as a complex network in which nodes represent distinct systems (e.g. brain and heart) and links, or edges, represent their coordination or synchronization, which can fluctuate depending on internal and external conditions (Ivanov 2021; Candia-Rivera et al. 2024). Edges are inferred from dynamical coupling within and between systems and sub-systems estimated from continuous, synchronous data streams of body and brain signals. Within this framework, methods such as mutual information, transfer entropy, and Granger causality offer alternative ways of operationalizing brain–body links, potentially providing principally new information beyond the correspondences reviewed above (Candia-Rivera et al. 2026). For example, Corcoran et al. (2025a. 2025b) found that HEPs and mutual information estimated between the instantaneous phase of cardiac activity and low-frequency EEG provided complementary information about task disengagement during a sustained attention task. Frontal and fronto-temporal late-latency HEPs were modulated before mind-wandering reports, but did not distinguish mind-blanking or vigilance level. By contrast, brain–heart mutual information decreased with task disengagement and declining vigilance.

Network analytic methods also enable analysis of statistical dependencies beyond two systems. Sitti et al. (2026) provisionally reported a resting-state midline central-occipital cluster wherein time-varying alpha power, cardiac autonomic indices, and normogastric power exhibited triadic multidirectional coupling at slow and infra-slow timescales, suggesting a coordinated brain–heart–gut network in which spontaneous fluctuations in one organ precede corresponding changes in other coupled systems.

#### 1.4.2 Binary hierarchy brain***–***body oscillation theory

As noted in the preceding sections, the binary hierarchy brain–body oscillation theory proposes that brain and bodily rhythms may be organized within a shared frequency architecture (Klimesch 2018). Frequency—the cycling of excitatory phases—may transiently accelerate or decelerate according to internal and external states (Cohen 2014; Samaha et al. 2015), but stable phase-locking can be achieved between two rhythms of varying timescales when their frequencies fluctuate such that they achieve an integer frequency ratio (e.g. r = fast/slow = integer; Palva and Palva 2018). The phenomenon, labeled cross-frequency synchronization (Palva and Palva 2018), is reflected latently in the center frequencies of the putative EEG and cardiorespiratory rhythms^3^: gamma (40 Hz), beta (20 Hz), alpha (10 Hz), theta (5 Hz), and delta (2.5 Hz) bands are related in integer multiples. Average resting heart rate is 1.25 Hz, aligning with delta, and the preferred breathing frequencies (0.3125, 0.1562, and 0.0781 Hz) are integer subdivisions of the heart rate.

Aforementioned findings include the enhanced coupling of alpha, heart rate, and breathing frequency during wakefulness relative to sleep (Rassi et al. 2019); and the enhanced coupling of alpha and heart rate in cognitive tasks relative to meditation (Soriano et al. 2024). Between brain sub-systems, it was found that coupling of alpha and theta bands increased linearly from meditative to mind wandering to cognitively demanding states (Rodriguez-Larios and Alaerts 2019, 2020, 2021). A recent study assessing cross-frequency synchronization between the five canonical EEG bands, heart rate, and breathing frequency found that this form of coupling was a systemic property of EEG and cardiorespiratory functional organization (Young et al. 2025). As a function of cognitive demand, task-related physiological arousal, and negative affect valence, cardiorespiratory rhythms coupled to network-level brain rhythms which in turn coupled to comparatively higher-frequency rhythms, suggestive of a hierarchical pattern of brain–body and intra-brain coordination. Consistent with the aforementioned hypothesis that heart–alpha cross-frequency synchronization reflects bodily awareness, coupling of heart rate and alpha was associated with a greater interoceptive sensitivity to increases in physiological arousal.

## 2 Pathology in the brain–body oscillatory hierarchy

We next articulate the second major point—that the rhythmic correspondences between brain and body systems, and the cognition they support, are vulnerable to dysfunction in the underlying bodily rhythm or, more importantly, the magnitude of the bodily rhythm’s modulation over brain processes. The coordination of brain and bodily processes is maintained through the ascending influence of bodily rhythms on neural activity, while these bodily rhythms, in turn, are subject to regulation by the central autonomic system. Bottom-up, the intersection of brain and bodily processes in interoception implicates atypical interoception in the pathology of neuropsychiatric disorders due to the functional overlap between interoception and, *inter alia*, sense of self in heart–brain coupling or vigilance in gastric–brain coupling (Khalsa et al. 2018; Brewer et al. 2021; Saltafossi et al. 2025). Top-down, the role of interoception in determining the perception of one’s own internal state implicates atypical interoception as an etiological factor in functional somatic disorders such as irritable bowel syndrome and chronic pain (Bonaz et al. 2021). The following will discuss, first, perturbations in the bodily rhythms themselves associated with disorder and, second, how the rhythmic correspondences reviewed above differ between healthy and distressed states, as well as between healthy and disordered populations.

### 2.1 Heart***–***brain dysfunction

Intrinsic heart rate, when severed from the brain, is higher than resting heart rate indicating that the central autonomic nervous system tonically inhibits cardiac activity (Thayer et al. 2012). Under acute threat, this inhibition is transiently released, allowing sympathetic activation to accelerate cardiac output. When threat detection becomes chronic, as in post-traumatic stress disorder, this state of disinhibition becomes chronic, contributing to sustained elevations in resting heart rate (Bedi and Arora 2007). Such sustained elevations are clinically consequential: resting heart rate independently predicts cardiovascular and all-cause mortality (Fox et al., 2007).

HRV decreases as heart rate accelerates (Sacha 2014). A healthy cardiovascular system is associated with high variability, a diseased system by comparatively less, and brain death by effectively none (Chalmers et al. 2014). An acceleration of heart rate and the related reduction in HRV reflects a release of inhibitory control by prefrontal systems as a consequence of psychogenic, biological, or environmental factors. Such autonomically inflexible states are associated with difficulty regulating emotion, anxio-depressive symptomatology (Schwerdtfeger et al. 2020), attenuated early-latency heartbeat-evoked potential (HEP; MacKinnon et al. 2013), and worse interoceptive accuracy (Lischke et al. 2021). Moreover, chronically elevated arousal has been suggested as a potential contributor to paranoid cognition, whereby physiological arousal is misinterpreted as fear and misattributed to nonexistent threats in the environment (Williams et al. 2004).

The heart is implicated as a modulator of interoceptive and affective processes. Its signals are sensitive to internal and external factors which may, top-down, become dysregulated by higher-level processes with reverberating consequences for related systems or, bottom-up, interfere with internally and externally oriented processing. The following will review how heart–brain coupling and the cognitive processes it is linked to may vary with adverse states and traits.

#### 2.1.1 Heartbeat-evoked potentials

Obsessive-compulsive (OCD) and anxiety disorders present with enhanced HEPs, suggesting a common theme of upregulated cortical gain on cardiac afferents that may contribute to symptom expression in these disorders. Overactive monitoring is well established in OCD (Endrass and Ullsperger 2014), and this overactive monitoring may extend to visceral signals. OCD patients outperform controls on heartbeat detection tasks and show greater early-to-mid HEP amplitude during both interoceptive and exteroceptive monitoring, despite poorer confidence and metacognitive awareness of their interoceptive performance (Yoris et al. 2017). This appears to manifest phenomenologically as greater subjective attention allocated to internal bodily sensations, greater worry about unpleasant sensations, and reduced bodily trust (Eng et al. 2020), motivating the conjecture that overactive visceral monitoring may underlie the sensory and visceral experiences that motivate compulsive behaviors (Bragdon et al. 2021).

Overactive monitoring is likewise implicated in anxiety disorders. For socially anxious individuals, interoceptive accuracy is associated with interaction anxiety and fear of negative evaluation, and high socially anxious individuals show more accurate heartbeat perception both at baseline and during speech anticipation, suggesting a trait-like tendency to monitor internal signals rather than a purely context-dependent response to social threat (Stevens et al. 2011). The overactive internal monitoring may become maladaptive when bodily arousal is interpreted as socially visible: high socially anxious individuals show enhanced early-to-mid HEP amplitude during false feedback of heart-rate acceleration, and this relationship is mediated by concerns about the social consequences of anxiety symptoms (Judah et al. 2018). In generalized anxiety disorder (GAD), altered HEP modulation suggests a more pervasive form of interoceptive hypervigilance. Whereas healthy controls show greater late-latency HEP amplitude during eyes-closed than eyes-open rest, GAD patients fail to show this context-dependent modulation because cardiac processing remains elevated during eyes-open rest, when interoceptive information is not task relevant (Pang et al. 2019). Both patterns align with meta-analytic evidence indicating that self-focused attention is associated with negative affect (Mor and Winquist 2002), suggesting that heightened internal monitoring may become maladaptive when bodily signals are recruited into a discrepancy process between one’s current embodied state and a desired or socially acceptable state.

Major depressive disorder shows a complementary but distinct pattern of interoceptive alteration: behaviorally, patients tend to perform worse on heartbeat-counting tasks (Dunn et al. 2007; Pollatos et al. 2009; Eggart et al. 2019), and cortically they exhibit attenuated HEPs across early, middle, and late latencies (Terhaar et al. 2012). This behavioral and cortical attenuation may index reduced sensitivity to visceral afferents, consistent with the broader flattening of affect and blunted motivational drive that characterize the disorder. Consistent with this interpretation, interoceptive accuracy (Herbert et al. 2011; Shah et al. 2016) and late-latency HEP amplitude (Rapp et al. 2025) are inversely associated with alexithymia, difficulty identifying and describing one’s own emotions (Bagby et al. 1994). Together, depression and related disorders may represent a decoupling of brain–body communication, wherein reduced cortical responsiveness to cardiac signals reflects a broader disintegration of the bidirectional brain–heart dialogue thought to underlie affective experience (Candia-Rivera 2022).

Beyond reflecting the content of subjective experience, cortical processing of interoceptive signals may also reflect the presence or dissolution of consciousness. In postcomatose patients, mid-to-late latency HEP amplitude predicts the presence of consciousness with greater accuracy than non-heartbeat-locked EEG signals (Candia-Rivera et al. 2021). Following this, heartbeat-evoked perturbational complexity, a measure of information richness (Casali et al. 2013) inspired by Integrated Information Theory (Tononi et al. 2016), was predictive for distinguishing those emerging from a minimally conscious state from those remaining in brain injury–related unconsciousness (Liuzzi et al. 2024). The predictive power afforded by HEPs—in magnitude and complexity—relative to non-heartbeat locked EEG suggests a functionally relevant contribution of visceral signals in generating subjective experience (Candia-Rivera 2022).

Our review of HEP dysfunction is certainly not comprehensive, but rather for the purposes of illustrating the adverse effect of brain–body coupling that is greater or lesser in magnitude relative to healthy controls, the effects of which may manifest phenomenologically as increased visceral salience to internal sensations or the sensation of disconnection from the body, respectively. This will be a recurring motif.

#### 2.1.2 Heart***–***alpha cross-frequency synchronization

Heart–alpha cross-frequency synchronization was previously found to increase as a function of cognitive demand (Soriano et al. 2024) and physiological arousal (Rassi et al. 2019). It was recently found that it was likewise associated with increases in self-reported fear-related emotions (Young et al. 2025). The more afraid one reported, the greater the coupling of alpha frequency and heart rate across the scalp. Unifying the three disparate but related findings, Young et al. (2025) conjectured that this form of brain–body coupling was associated with increases in state stress of which cognitive demand, physiological arousal, and negatively valenced affect were constituent elements, suggesting bodily awareness varies according to state- and trait-related stress. Consistent with this view, it was found that heart–alpha cross-frequency synchronization was linked to trait-related differences in subjective sensitivity to increases in physiological arousal (Young et al. 2025).

#### 2.1.3 HRV***–***neuroelectric phase***-***amplitude coupling

Prior studies found low- and high-frequency HRV phase to modulate the amplitude of neuroelectric oscillations (Sargent et al. 2024; Liao et al. 2025; Xiong et al. 2025), with the former linked to emotional control and the latter conjectured to reflect emotion regulation. Motivated by evidence linking schizophrenia and autonomic dysfunction, Sargent et al. (2025) found high-frequency HRV phase-amplitude coupling of fronto-central alpha and theta bands to be lower in schizophrenia. Such differences in brain–body coupling were able to distinguish the schizophrenic sample from healthy controls beyond HRV and EEG alone. Notably, Granger causality analyses suggested that this coupling retained heart-to-brain directionality in both groups. Sargent et al. (2025) interpreted the findings as indicative of a breakdown in the body’s role as a binding agent for functionally integrating cortical processes across spatiotemporal scales.

Low-frequency HRV phase was found to modulate alpha-band amplitude in bilateral insula and left entorhinal cortex in a healthy sample (Liao et al. 2025). In major depressive disorder, Liao et al. (2025) reported a more extensive network of phase-amplitude coupling between low-frequency HRV and alpha amplitude, including the bilateral pars triangularis and left pars orbitalis, as well as significantly stronger coupling in the left insula. Coupling within the left insula scaled with depression severity. The authors interpreted this pattern as reflecting a compensatory mechanism for weakened inhibitory control over sympathetic activity. Increased bottom-up, low-frequency modulation of the insula and the recruitment of regions involved in emotion processing may reflect an attempt to regulate the internal state in the face of chronic anticipation of stressors, though this interpretation is provisional.

### 2.2 Breath***–***brain dysfunction

Across clinical populations, characteristic disturbances in respiration are common (Heck et al. 2022; Brændholt et al. 2023). In schizophrenia, patients breathe faster, more shallowly, and more variably relative to healthy controls—the variability of which is correlated with positive symptoms (Bär et al. 2012). Schizophrenic patients fail to adjust breathing to present context: healthy controls slow and deepen their breathing under pleasant stimuli and accelerate respiration under white noise, but patients breathe rapidly and shallowly across all conditions (Akar et al. 2012). That breathing abnormalities are detectable across conditions and scale with positive symptomatology suggests this reflects autonomic dysregulation with functional consequences for related systems, such as the heart, through the respiratory sinus arrhythmia. Indeed, patients with schizophrenia exhibit comparatively high prevalence for cardiovascular risk factors, conjectured to be (partly) responsible for the reduced life expectancy associated with the disorder (Polcwiartek et al. 2025). Respiration in autism spectrum disorder is likewise characterized by irregular, shallow breaths of varying depth followed by unpredictable periods of breathing cessation (Ming et al. 2016) and, also like atypical breathing in schizophrenia, autistic individuals suffer from autonomic dysregulation (Bujnakova et al. 2016).

It is well known that hyperventilation is a core feature of panic attacks (Sinha et al. 2000). A substantial proportion of individuals with panic attacks present with a respiratory subtype marked by prominent dyspneic symptoms (breathlessness), greater carbon dioxide sensitivity, and a disproportionate fear of suffocation (Goodwin et al. 2002; Sardinha et al. 2009). Experimental inductions of dyspnea cause individuals both high in anxiety sensitivity and suffocation fear to experience greater air hunger, greater anxiety, and maladaptive increases in ventilation that paradoxically worsen their discomfort (Alius et al. 2013). At baseline, patients exhibit more variable respiration relative to healthy controls (Grassi et al. 2013) and increases in respiratory variability precede panic attack onset (Meuret et al. 2011). Heck et al. (2022) argue such fluctuations in variability in the breathing patterns of individuals with panic disorder likely propagate to the limbic system through respiration-locked neural oscillations, thereby enhancing the salience of air hunger.

Respiration-locked oscillations depend on initial oscillatory activity in the olfactory bulb (Brændholt et al. 2023). Anosmics (loss of sense of smell) and normosmics breathe at a similar respiratory rate, but anosmics exhibit markedly different nasal airflow: they produce fewer inhalation peaks during wakefulness, more inhalation pauses, reduced exhalation peak flow, and increased variability of inhalation volume during sleep (Gorodisky et al. 2024). Consistent with the role of olfactory bulb–mediated respiration-locked oscillations in modulating limbic activity, congenital and acquired anosmia are associated with depression (Pollatos et al. 2007; Kohli et al. 2016), personal isolation, and emotional blunting (Toller 1999). Relatedly, although individuals high in alexithymia subjectively rate odors similarly to those low in alexithymia, they exhibit exaggerated yet undifferentiated physiological arousal across unpleasant, neutral, and even clean-air stimuli (Cecchetto et al. 2017).

Respiratory components such as rate, variability, and waveform are sensitive to disorder and can themselves serve as indices of pathology. The following will review the present research on dysfunction in breath–brain coupling.

#### 2.3 Breath***–***brain neuroelectric coupling

The breath coordinates sensory sampling to the excitability of internal networks. In a focal epilepsy case report, the influence of respiration on cortical excitability was found to play a direct role in the timing and emergence of epileptiform activity (Kluger et al. 2025). Healthy controls exhibited decreased cortical excitability (sharper 1/f slope) at expiration-to-inspiration transitions and increased cortical excitability at inspiration-to-expiration transitions (flatter 1/f slope), but the focal epilepsy patient exhibited increased cortical excitability at expiration-to-inspiration transitions as well as at inspiration-to-expiration transitions. Respiration-locked increases in excitability occurred twice as often in the patient. Critically, interictal epileptiform discharges—transient hyper-synchronization of cortical neurons between seizures—occurred preferentially during respiratory phase transitions and related periods of upregulated cortical excitability.

The case report suggests respiration can aberrantly modulate excitation–inhibition balance toward states of hyper-excitability (Kluger et al. 2025). It raises the question of how respiration might influence the excitation–inhibition balance in disorders where this balance differs from that of healthy controls, such as autism spectrum disorder (Rubenstein and Merzenich 2003)—particularly given that autism is associated with dysrhythmic breathing (Ming et al. 2016).

### Gastric***–***brain dysfunction

The phylogenetic trajectory shared by the enteric nervous system and state regulation is strongly implicated in the robust comorbidity between socioemotional and gastrointestinal disorders (Kolacz et al. 2019; Margolis et al. 2021). Much of this literature has emphasized microbiota-mediated communication between the gastrointestinal and central nervous systems (Person and Keefer 2021), but recent findings pertaining to the oscillatory coupling of the gastric basal rhythm to neuroelectric (Richter et al. 2017) and neurovascular signals (Rebollo et al. 2018) have prompted scholars to consider these rhythmic correspondences as viable pathways by which gastric–brain communication may occur more rapidly and directly than previously assumed (Palacios-García and Parada 2020).

In healthy adults, gastric rhythms range from 0.025 to 0.060 Hz (normogastria), but may become dysrhythmic, atypically decelerated (bradygastria) or accelerated (tachygastria), by biological and psychogenic causes. In inflammatory bowel disease, gastric myoelectrical activity is altered even when disease is clinically remitted: patients with ulcerative colitis exhibit reduced resting dominant frequency consistent with bradygastria (Sharma et al. 2015), whereas patients with Crohn’s disease show increased bradygastric and tachygastric activity (Kohno et al. 2006). Functional gastrointestinal disorders, such as irritable bowel syndrome, which are not attributable to organic explanation (Black et al. 2020) are likewise characterized by gastric dysrhythmia (Mazur et al. 2007). Importantly, gastric dysrhythmia is also reported in major depressive disorder in the absence of gastrointestinal disorder (Ruhland et al. 2008; Quick et al. 2010).

A discussion of gastric dysrhythmia is relevant because gastric frequency covaries with gastric interoception. Lower normogastria has been linked to less accurate perception of comfortable satiety and fullness thresholds in emotional eating (Tiemann et al. 2025). Conversely, bradygastria is associated with altered gastric interoception: individuals with binge-eating pathology show reduced normogastric power, increased bradygastric power, and delayed satiation during water-load testing, suggesting that bradygastria may be associated with less precise interoceptive signals (van Dyck et al. 2021). Tachygastria, by contrast, appears linked to aversive gastric salience. When motion-sick, tachygastria is a strong correlate of symptom severity, including nausea and stomach discomfort (Hu et al. 1999). Similarly, experienced core disgust is more strongly related to tachygastria than is body-boundary-violation disgust (Harrison et al. 2010), and pharmacologically attenuating gastric dysrhythmia reduces oculomotor disgust avoidance following incentivized exposure (Nord et al. 2021). Thus, gastric frequency may covary with interoception along a salience gradient: bradygastria appears linked to imprecise gastric interoception, whereas tachygastria appears linked to aversively salient gastric interoception.

The theoretical importance of these frequency-specific interoceptive profiles lies in the dependence of gastric–brain coupling on gastric phase. Because phase evolves as a function of frequency over time, bradygastric and tachygastric rhythms necessarily alter the temporal trajectory of the gastric signal. Existing gastric–brain coupling studies have primarily characterized coupling to resolved normogastric rhythms in healthy participants, including gastric–alpha phase-amplitude coupling (Richter et al. 2017) and gastric-BOLD phase-locking (Rebollo et al. 2021), both interpreted as involving predominantly ascending gastric-to-brain directionality. In these studies, normogastric specificity indicates that neural correspondence tracks the individual’s gastric rhythm. Whether coupling is absent, preserved, or reorganized outside the normogastric range in dysrhythmic populations or states remains an open question. The conservative implication is that gastric dysrhythmia, which covaries with interoceptive and affective states, alters the temporal structure of a bodily signal that is both interoceptively consequential and capable of organizing neural activity.

#### 2.3.1 Gastric–neuroelectric phase-amplitude coupling

In a pilot study, Jeanne et al. (2023) found acute stress increases phase-amplitude coupling (PAC) of gastric phase and alpha amplitude in fronto-temporal channels. The authors interpreted this enhancement of PAC under stress as increased cortical entrainment to gastric signals, reflecting greater attention toward visceral signals. Such state-level, stress-induced increases in bodily awareness are consistent with evidence of enhanced interoception following social stressors (Maeda et al. 2019) and acute exercise (Wallman-Jones et al. 2021).

At the trait level, gastric interoception is associated with the valence of one’s body image. The more sensitive one is to gastric sensations (e.g. satiety cues), the greater their responses to positive body image questionnaires (Todd et al. 2020) and, conversely, the less sensitive one is, the more negative their body image^4^ and the greater their reported body image disturbances (Van Dyck et al. 2016). Todd et al. (2021) found that individual differences in gastric–alpha PAC in left centroparietal channels were inversely associated with body shame and weight preoccupation. The lack of association with positive body image measures is notable, suggesting that relatively weak gastric–alpha PAC may index an implicit vulnerability to negative, affectively charged body representations. Weaker stomach–brain coupling may reflect weaker interoceptive contributions to bodily self-representation, thereby necessitating that the individual use exteroceptive cues as the basis for their bodily awareness resulting in a disproportionate focus on the aesthetic characteristics of the body (Todd et al. 2021).

#### 2.3.2 Gastric-BOLD phase-locking

Banellis et al. (2025) found gastric-BOLD phase-locking to index self-reported mental health such that the higher an individual’s reported anxiety, depression, fatigue, and stress, the greater their gastric–brain coupling in frontoparietal regions. A canonical variate was identified on which negative mental health items including anxiety, depression, fatigue, and stress load negatively, and positive mental health items including well-being and quality of life load positively. This variate was inversely associated with gastric-BOLD phase-locking in dorsal attention, frontoparietal control, ventral salience, and somatomotor networks—systems involved in orienting, executive control, interoceptive–affective integration, and bodily representation. The findings suggest that psychological distress is associated with stronger coupling between the gastric basal rhythm and BOLD fluctuations (Banellis et al. 2025).

Levakov et al. (2021) identified a resting-state subnetwork—predominantly sensory-motor and visual cortices—whose baseline connectivity predicted subsequent weight loss and which spatially overlapped with the aforementioned gastric network (Rebollo and Tallon-Baudry 2022). Critically, within this subnetwork, BOLD power at the normogastric frequency was inversely associated with future weight loss, and this association was specific both to normogastric frequency and to the subnetwork itself. The authors frame this in the context of over-sensitivity to food cues and propose a plausible gastric–brain coupling mechanism—while noting it is correlational and *post hoc*. Taken together, increases in coupling of BOLD to gastric rhythms^5^ are associated with maladaptive interoception that may morph the perception of one’s body and impair homeostasis.

## 3. Discussion

If the proverbial brain-in-a-vat were possible, what would it be missing? The evidence reviewed herein suggests that it would lack not merely a stream of bodily inputs, but the structured dialogue with the body that supplies the visceral dimension of phenomenological experience and the mechanisms by which disparate neural systems may coordinate their activity. We have argued, first, that interoception and functionally related cognition—including, *inter alia*, emotion, selfhood, and perception—are supported by structured rhythmic correspondences between brain and bodily rhythms. The bodily rhythms—heartbeat, breath, and gastric basal rhythm—are, in and of themselves, representations of the organism’s state, reflecting its health (e.g. gastric dysrhythmia from gut inflammation) and function (e.g. accelerated heart rate by sympathetic activation). These rhythms influence neural dynamics in two ways. They convey information about the bodily state in support of interoception and related cognition and provide a shared temporal structure by which distinct cognitive and sensory systems may coordinate their respective processes.

We argued, second, that the functional role of brain–body synchrony is most salient in states of disorder. The reviewed clinical and subclinical conditions indicate that disturbances in these cognitive and affective functions are associated with both alterations in bodily rhythm parameters (e.g. rate) and changes in the magnitude of those rhythms’ modulation of brain processes, with the latter sometimes varying independently of the underlying bodily rhythm itself (e.g. Banellis et al. 2025). Observations within this latter portion of our review motivate two ancillary conjectures.

First, there exists an optimal range of brain–body coupling, beyond which greater and lesser coupling is associated with adverse effects. Greater coupling of brain and body rhythms may over-weight bodily signals in internal models, such that transient perturbations in heart, breath, or gut are catastrophically interpreted as evidence of imminent threat or bodily failure, precipitating anxious hypervigilance or compulsive checking. Less coupling, in turn, may reduce the precision of interoception, weakening the felt presence of the body as a reference frame and contributing to emotional numbing, depersonalization, or alienation from one’s own bodily state.

Second, the data suggest an emergent pattern whereby indices of brain–body synchrony often increase as a function of stress—notably, state- or task-related stress. Cross-frequency synchronization between EEG and cardiorespiratory rhythms increases with task-related stress (Young et al. 2025). Gastric-alpha phase-amplitude coupling increases under acute stress induction (Jeanne et al. 2023). Gastric-BOLD phase-locking increases with self-reported anxiety, depression, fatigue, and stress^6^ (Banellis et al. 2025). Fast breathing, which covaries with stress and anxiety (Engelen et al. 2023), induces stronger respiration-locked modulation of cortical activity (Herrero et al. 2018). That the brain under demand increasingly couples to the heart and breath is intuitive, as both are upregulated by sympathetic activation and support fight-or-flight behaviors. The tension arises in the observation that coupling to the stomach likewise increases, despite acute stress–related suppression of gastric motility (Taché and Bonaz 2007) and perturbation of myoelectrical activity in and around the normogastric range (Huerta-Franco et al. 2012). A compelling conjecture, to reconcile this tension, is that the brain under duress is not simply promoting interoceptive signals to increase interoceptive awareness—which may be of limited contextual advantage—but rather to harness those signals as a shared temporal scaffold for coordinating multiple cognitive systems. In this view, enhanced brain–body coupling under stress serves to align distributed networks around the common temporal structure supplied by cardiac, respiratory, and gastric rhythms, thereby facilitating multisensory integration, vigilance, and other forms of integrated cognition. It remains an empirical question whether brain–body synchrony enhancements under state- or task-related stress primarily increase interoceptive awareness, facilitate the coordination of distributed cognitive systems *via* a shared temporal scaffold, or negotiate a context-dependent balance between these functions.

We approached consciousness indirectly,^7^ by proxy of cognitive and affective functions thought to index the presence, content, and quality of phenomenological experience. Our first major point of discussion argued that these faculties are supported by various forms of brain–body synchrony. Our second major point argued that dysfunction in these faculties often coincides with dysfunction in brain–body synchrony. This brings us to our third and last major point: consciousness and its constellatory constructs are inseparable from their embodiment. When underlying bodily rhythms become dysrhythmic, or when the brain and body, in simple terms, become over- or under-coupled, the constellation is reconfigured in ways that map onto clinical phenomenology. This marks a categorical distinction between the vatted and embodied brains.

In closing, as other scholars have noted (Kluger et al. 2024), human neuroscience is experiencing an evolution in perspective. It is the recognition that cognition is an object more complex for its distributed, multivariate nature, yet more simple because it is constrained by the nervous system’s interactions with its environment. When asked to point to where you—the conscious agent—are located, the common response is to indicate a place behind the eyes because vision is the most prominent of our senses. Historically, however, it was the heart that was presumed to be the seat of consciousness for the strong visceral nature of its function (Jaynes 1976; Cobb 2020). There is an emerging middle ground perspective that identifies the two as one and the same systems whereby action, health, and disease may be represented as organismal phenomena.

### 3.1 Transdiagnostic biotypes

One promising future direction is to leverage brain–body synchrony as a basis for identifying transdiagnostic biotypes of neuropsychiatric disorder (Banellis et al. 2025). The present review suggests that different axes of rhythmic correspondence between brain and body track clinical symptomatology such as hypervigilance, dysphoria, dissociation, and disturbed body image across diagnostic categories. Individuals, states, or disorders may be characterized by multivariate profiles of heart–, breath–, and gastric–brain coupling at rest and under controlled perturbations. Such biotypes may ultimately guide mechanism-based interventions targeting specific rhythmic correspondences *via* the underlying bodily rhythm (e.g. Xiong et al. 2025) or *via* vagus nerve projections that innervate the generators of these rhythms (e.g. Müller et al. 2022).

### 3.2 An integrated biopsychosocial model of embodiment

We have, for the duration of this paper, discussed brain–body synchrony and embodied cognition at the organismal scale. Humans, however, are social animals and the embodiment of our cognition extends to the intersubjective environment (Thompson and Varela 2001). The brain regions responsible for representing the self, particularly those that represent the bodily state, overlap considerably with those regions responsible for representing others such that it has been conjectured that the self cannot be grasped independently of a conceptualization of others (Decety and Sommerville 2003). Consistent with this view, interoception and empathy appear to be linked. Greater accuracy in detecting one’s heartbeat is associated with higher estimated pain intensity and greater reported compassion when viewing another in pain (Grynberg and Pollatos 2015), as well as increased monetary generosity in the dictator-game (Piech et al. 2017). At the neural level, HEP amplitude correlates with self-reported empathic concern and is dynamically modulated during tasks involving explicit empathic processing (Fukushima et al. 2011).

It has been demonstrated that humans can interpersonally synchronize behavior, physiology, and brains, and this synchrony corresponds to enhanced performance in cooperative tasks (Hasson et al. 2012; Dikker et al. 2017; Nguyen et al. 2020), homophily and social network proximity (Parkinson et al. 2018), experiences of self–other merging (Valencia and Froese 2020), and increased feelings of group affiliation (Hove and Risen 2009; Hoehl et al. 2021).

Coupling at the organismal scale—between the brain and body—foundationally supports interoception and functionally related cognition, including the perception and modeling of others. Coupling at the interpersonal scale—between distinct individuals—enhances the group experience and cooperative ability, giving rise to the conjecture that consciousness and cognition may indeed extend beyond not just the brain, but the organism itself (Valencia and Froese 2020; Young et al. 2022b). Together, these nested layers of coupling—internal and interpersonal—form the basis of a true biopsycho*social* model of embodiment whereby cognition is nested within an enactive physiology and the brain–body system is nested within a dynamic world.

## Footnotes

1 A useful, though imperfect, model of the disembodied brain is provided by hemispherotomy: a surgical procedure in which an epileptogenic hemisphere is disconnected from interhemispheric, subcortical, and sensorimotor pathways while remaining vascularized in the cranial cavity. Isolated hemispheres can retain some intrinsic network organization, yet available fMRI and EEG evidence suggests activity patterns associated with reduced consciousness, including lower consciousness-classifier scores and persistent sleep-like slow waves in awake patients (Blauwblomme et al. 2020; Colombo et al. 2025).

2 Also referred to as the pulsed inhibition hypothesis (Engelen et al. 2023).

3 It is worth noting that gastric rhythms are omitted from this framework. The 0.05-Hz gastric basal rhythm does not follow the integer frequency structure of the EEG and cardiorespiratory rhythms, though this reconciled within the theory by proposing that gastric rhythms are a constituent element of a separate functional hierarchy akin to the popular divisions of fight-or-flight and rest-and-digest groupings of cardiorespiratory and gastrointestinal systems (Klimesch 2018).

4 Positive (e.g. body appreciation, functionality appreciation) and negative (e.g. body shame, weight preoccupation) body image are distinct constructs (Todd et al. 2021).

5 Insofar as the Levakov et al. (2021) findings may suggest a gastric-BOLD coupling mechanism.

6 The canonical variate representing mental health that was inversely related to gastric-BOLD coupling was composed of both state (e.g. Patient Health Questionnaire-9) and trait questionnaires (e.g. State–Trait Anxiety Inventory).

7 In some cases directly, as demonstrated in HEP components indexing the presence of consciousness in comatose patients (Candia-Rivera et al. 2021; Liuzzi et al. 2024).
